# Diaqua­bis(cyclo­hexa­necarboxyl­ato)zinc(II) monohydrate

**DOI:** 10.1107/S1600536809033479

**Published:** 2009-08-29

**Authors:** Yue-Wu Zhang, Li-Jun Wang, Jian Hou, Qing-Fu Zeng

**Affiliations:** a Engineering Research Center for Clean Production of Textile Dyeing and Printing, Ministry of Education, Wuhan 430073, People’s Republic of China

## Abstract

In the title compound, [Zn(C_7_H_11_O_2_)_2_(H_2_O)_2_]·H_2_O, the Zn^II^ atom (site symmetry 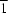) is four-coordinated by two O atoms from the cyclo­hexa­necarboxyl­ate anions and two O atoms from the water mol­ecules, forming a slightly distorted square-planar coordination. The O atom of the uncoordinated water mol­ecule lies on a crysatllographic twofold rotation axis. In the crystal, the components are linked by O—H⋯O hydrogen bonds, forming a three-dimensional network.

## Related literature

For background, see: Cheng *et al.* (2006 ▶). For reference structural data, see: Allen *et al.* (1987 ▶).
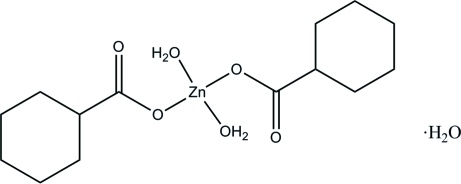


## Experimental

### 

#### Crystal data

[Zn(C_7_H_11_O_2_)_2_(H_2_O)_2_]·H_2_O
*M* *_r_* = 373.75
Monoclinic, 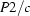

*a* = 15.9045 (16) Å
*b* = 4.9295 (6) Å
*c* = 11.5335 (16) Å
β = 91.585 (6)°
*V* = 903.89 (19) Å^3^
*Z* = 2
Mo *K*α radiation
μ = 1.39 mm^−1^
*T* = 296 K
0.28 × 0.25 × 0.22 mm

#### Data collection

Enraf–Nonius CAD-4 diffractometer
Absorption correction: ψ scan (North *et al.*, 1968 ▶) *T*_min_ = 0.698, *T*_max_ = 0.750
4764 measured reflections
1752 independent reflections
1436 reflections with *I* > 2σ(*I*)
*R*_int_ = 0.032
200 standard reflections every 3 reflections intensity decay: 1%

#### Refinement

*R*[*F* ^2^ > 2σ(*F* ^2^)] = 0.054
*wR*(*F* ^2^) = 0.158
*S* = 1.07
1752 reflections
114 parameters
4 restraints
H atoms treated by a mixture of independent and constrained refinement
Δρ_max_ = 1.00 e Å^−3^
Δρ_min_ = −0.37 e Å^−3^

### 

Data collection: *CAD-4 Software* (Enraf–Nonius, 1989 ▶); cell refinement: *CAD-4 Software*; data reduction: *XCAD4* (Harms & Wocadlo, 1995 ▶); program(s) used to solve structure: *SHELXS97* (Sheldrick, 2008 ▶); program(s) used to refine structure: *SHELXL97* (Sheldrick, 2008 ▶); molecular graphics: *SHELXTL* (Sheldrick, 2008 ▶); software used to prepare material for publication: *SHELXTL*.

## Supplementary Material

Crystal structure: contains datablocks global, I. DOI: 10.1107/S1600536809033479/hb5057sup1.cif

Structure factors: contains datablocks I. DOI: 10.1107/S1600536809033479/hb5057Isup2.hkl

Additional supplementary materials:  crystallographic information; 3D view; checkCIF report

## Figures and Tables

**Table 1:** Selected bond lengths (Å)

Zn1—O2	1.961 (3)
Zn1—O3	1.977 (4)

**Table 2:** Hydrogen-bond geometry (Å, °)

*D*—H⋯*A*	*D*—H	H⋯*A*	*D*⋯*A*	*D*—H⋯*A*
O7—H7⋯O1	0.834 (10)	1.99 (3)	2.699 (4)	142 (5)
O3—H3*D*⋯O1^i^	0.846 (10)	2.53 (7)	3.132 (6)	129 (8)
O3—H3*C*⋯O7^ii^	0.853 (10)	2.17 (2)	2.997 (5)	164 (6)

Symmetry codes: (i) 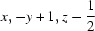; (ii) 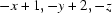.

**Fig. 1.**
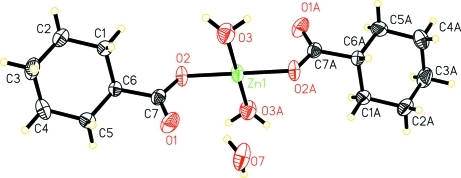
The molecular structure of (I) showing 30% probability displacement ellipsoids. Atoms with the suffix A are generated by the symmetry code (1–x, 1–y, –z).

## supplementary crystallographic information

### Comment

There has been much research interest in acid metal complexes due to their molecular architectures and biological activities (e.g. Cheng *et al.*, 2006). In this work, we report here the crystal structure of the title compound, (I). In (I), all bond lengths are within normal ranges (Allen *et al.*, 1987) (Fig. 1). The Zn^II^ atom is four-coordinated by two O atoms from the cyclohexanecarboxylate and two O atoms from the water molecules, forming a slightly distorted square-planar coordination (Table 1).  In the crystal, O—H···O hydrogen bonds (Table 2) link the components.

### Experimental

A mixture of cyclohexanecarboxylic acid (256 mg, 2 mmol) and ZnNO_3_.6H_2_O (1 mmol, 297 mg) in methanol (10 ml) was stirred for 2 h. After keeping the filtrate in air for 6 d, colourless blocks of (I) were formed.

### Refinement

The O-bound H atoms were located in a difference map and their positions were refined with the restraint O—H = 0.82 (1)Å. The other H atoms were positioned geometrically (C—H = 0.97–0.98Å) and refined as riding, with *U*_iso_(H) = 1.2*U*_eq_(carrier).

### Crystal data

**Table:**

[Zn(C_7_H_11_O_2_)_2_(H_2_O)_2_]·H_2_O	*F*(000) = 396
*M**_r_* = 373.75	*D*_x_ = 1.373 Mg m^−^^3^
Monoclinic, *P*2/*c*	Mo *K*α radiation, λ = 0.71073 Å
Hall symbol: -P 2yc	Cell parameters from 25 reflections
*a* = 15.9045 (16) Å	θ = 9–12°
*b* = 4.9295 (6) Å	µ = 1.39 mm^−^^1^
*c* = 11.5335 (16) Å	*T* = 296 K
β = 91.585 (6)°	Block, colorless
*V* = 903.89 (19) Å^3^	0.28 × 0.25 × 0.22 mm
*Z* = 2	

### Data collection

**Table:**

Enraf–Nonius CAD-4 diffractometer	1436 reflections with *I* > 2σ(*I*)
Radiation source: fine-focus sealed tube	*R*_int_ = 0.032
graphite	θ_max_ = 26.0°, θ_min_ = 2.6°
ω/2θ scans	*h* = −18→19
Absorption correction: ψ scan (North *et al.*, 1968)	*k* = −6→6
*T*_min_ = 0.698, *T*_max_ = 0.750	*l* = −12→14
4764 measured reflections	200 standard reflections every 3 reflections
1752 independent reflections	intensity decay: 1%

### Refinement

**Table:**

Refinement on *F*^2^	Primary atom site location: structure-invariant direct methods
Least-squares matrix: full	Secondary atom site location: difference Fourier map
*R*[*F*^2^ > 2σ(*F*^2^)] = 0.054	Hydrogen site location: inferred from neighbouring sites
*wR*(*F*^2^) = 0.158	H atoms treated by a mixture of independent and constrained refinement
*S* = 1.07	*w* = 1/[σ^2^(*F*_o_^2^) + (0.0809*P*)^2^ + 1.216*P*] where *P* = (*F*_o_^2^ + 2*F*_c_^2^)/3
1752 reflections	(Δ/σ)_max_ = 0.003
114 parameters	Δρ_max_ = 1.00 e Å^−^^3^
4 restraints	Δρ_min_ = −0.37 e Å^−^^3^

### Special details

**Table:**

Geometry. All e.s.d.'s (except the e.s.d. in the dihedral angle between two l.s. planes) are estimated using the full covariance matrix. The cell e.s.d.'s are taken into account individually in the estimation of e.s.d.'s in distances, angles and torsion angles; correlations between e.s.d.'s in cell parameters are only used when they are defined by crystal symmetry. An approximate (isotropic) treatment of cell e.s.d.'s is used for estimating e.s.d.'s involving l.s. planes.
Refinement. Refinement of *F*^2^ against ALL reflections. The weighted *R*-factor *wR* and goodness of fit *S* are based on *F*^2^, conventional *R*-factors *R* are based on *F*, with *F* set to zero for negative *F*^2^. The threshold expression of *F*^2^ > σ(*F*^2^) is used only for calculating *R*-factors(gt) *etc*. and is not relevant to the choice of reflections for refinement. *R*-factors based on *F*^2^ are statistically about twice as large as those based on *F*, and *R*- factors based on ALL data will be even larger.

### Fractional atomic coordinates and isotropic or equivalent isotropic displacement parameters (Å^2^)

**Table:**

	*x*	*y*	*z*	*U*_iso_*/*U*_eq_	
C1	0.2156 (3)	0.3294 (12)	−0.0060 (4)	0.0586 (12)	
H1A	0.2463	0.2824	−0.0747	0.070*	
H1B	0.2126	0.5256	−0.0017	0.070*	
C2	0.1273 (3)	0.2162 (13)	−0.0177 (4)	0.0711 (14)	
H2A	0.0988	0.2966	−0.0847	0.085*	
H2B	0.1301	0.0218	−0.0300	0.085*	
C3	0.0779 (3)	0.2734 (13)	0.0889 (5)	0.0743 (15)	
H3A	0.0709	0.4676	0.0980	0.089*	
H3B	0.0226	0.1917	0.0810	0.089*	
C4	0.1236 (3)	0.1592 (16)	0.1934 (5)	0.0832 (18)	
H4A	0.1264	−0.0367	0.1864	0.100*	
H4B	0.0925	0.2017	0.2622	0.100*	
C5	0.2124 (3)	0.2720 (13)	0.2071 (4)	0.0661 (14)	
H5A	0.2095	0.4653	0.2221	0.079*	
H5B	0.2405	0.1867	0.2733	0.079*	
C6	0.2630 (2)	0.2234 (9)	0.0999 (3)	0.0427 (9)	
H6	0.2708	0.0274	0.0908	0.051*	
C7	0.3485 (2)	0.3560 (8)	0.1063 (4)	0.0455 (9)	
H7	0.475 (3)	0.722 (8)	0.205 (3)	0.058 (14)*	
O1	0.3660 (3)	0.5302 (7)	0.1802 (4)	0.0755 (11)	
O2	0.39946 (17)	0.2822 (6)	0.0283 (3)	0.0549 (8)	
O3	0.4282 (3)	0.7551 (8)	−0.0904 (4)	0.0718 (10)	
O7	0.5000	0.8302 (10)	0.2500	0.0775 (16)	
Zn1	0.5000	0.5000	0.0000	0.0492 (3)	
H3D	0.384 (3)	0.71 (2)	−0.127 (5)	0.18 (5)*	
H3C	0.457 (3)	0.852 (11)	−0.136 (4)	0.076 (18)*	

### Atomic displacement parameters (Å^2^)

**Table:**

	*U*^11^	*U*^22^	*U*^33^	*U*^12^	*U*^13^	*U*^23^
C1	0.045 (2)	0.085 (4)	0.046 (2)	−0.007 (2)	−0.0019 (18)	0.005 (2)
C2	0.048 (3)	0.097 (4)	0.067 (3)	−0.012 (3)	−0.011 (2)	−0.006 (3)
C3	0.037 (2)	0.094 (4)	0.092 (4)	−0.003 (2)	0.000 (2)	−0.010 (3)
C4	0.057 (3)	0.120 (5)	0.075 (3)	−0.017 (3)	0.026 (3)	0.004 (4)
C5	0.053 (3)	0.101 (4)	0.045 (2)	−0.002 (3)	0.005 (2)	0.002 (2)
C6	0.0374 (19)	0.046 (2)	0.045 (2)	−0.0065 (16)	0.0017 (16)	−0.0002 (17)
C7	0.041 (2)	0.041 (2)	0.054 (2)	−0.0016 (17)	−0.0066 (18)	0.0067 (19)
O1	0.065 (2)	0.074 (3)	0.086 (3)	−0.0155 (18)	−0.0144 (19)	−0.0217 (19)
O2	0.0323 (14)	0.0504 (17)	0.083 (2)	−0.0060 (12)	0.0125 (14)	0.0045 (15)
O3	0.060 (2)	0.066 (2)	0.089 (3)	−0.0020 (18)	−0.004 (2)	0.012 (2)
O7	0.047 (3)	0.041 (3)	0.143 (5)	0.000	−0.008 (3)	0.000
Zn1	0.0317 (4)	0.0478 (4)	0.0684 (5)	−0.0007 (3)	0.0066 (3)	0.0097 (3)

### Geometric parameters (Å, °)

**Table:**

C1—C6	1.511 (6)	C5—H5A	0.9700
C1—C2	1.513 (6)	C5—H5B	0.9700
C1—H1A	0.9700	C6—C7	1.509 (5)
C1—H1B	0.9700	C6—H6	0.9800
C2—C3	1.503 (7)	C7—O1	1.236 (5)
C2—H2A	0.9700	C7—O2	1.281 (5)
C2—H2B	0.9700	Zn1—O2	1.961 (3)
C3—C4	1.500 (8)	Zn1—O3	1.977 (4)
C3—H3A	0.9700	O3—H3D	0.846 (10)
C3—H3B	0.9700	O3—H3C	0.853 (10)
C4—C5	1.521 (7)	O7—H7	0.834 (10)
C4—H4A	0.9700	Zn1—O2^i^	1.961 (3)
C4—H4B	0.9700	Zn1—O3^i^	1.977 (4)
C5—C6	1.513 (6)		
			
C6—C1—C2	112.6 (4)	C6—C5—H5A	109.3
C6—C1—H1A	109.1	C4—C5—H5A	109.3
C2—C1—H1A	109.1	C6—C5—H5B	109.3
C6—C1—H1B	109.1	C4—C5—H5B	109.3
C2—C1—H1B	109.1	H5A—C5—H5B	107.9
H1A—C1—H1B	107.8	C7—C6—C1	108.6 (3)
C3—C2—C1	111.3 (4)	C7—C6—C5	113.0 (4)
C3—C2—H2A	109.4	C1—C6—C5	110.0 (4)
C1—C2—H2A	109.4	C7—C6—H6	108.4
C3—C2—H2B	109.4	C1—C6—H6	108.4
C1—C2—H2B	109.4	C5—C6—H6	108.4
H2A—C2—H2B	108.0	O1—C7—O2	123.1 (4)
C4—C3—C2	109.5 (4)	O1—C7—C6	121.4 (4)
C4—C3—H3A	109.8	O2—C7—C6	115.5 (4)
C2—C3—H3A	109.8	C7—O2—Zn1	119.8 (3)
C4—C3—H3B	109.8	Zn1—O3—H3D	123 (6)
C2—C3—H3B	109.8	Zn1—O3—H3C	112 (4)
H3A—C3—H3B	108.2	H3D—O3—H3C	107.5 (17)
C3—C4—C5	112.0 (5)	O2—Zn1—O2^i^	180.0
C3—C4—H4A	109.2	O2—Zn1—O3	88.54 (15)
C5—C4—H4A	109.2	O2^i^—Zn1—O3	91.46 (15)
C3—C4—H4B	109.2	O2—Zn1—O3^i^	91.46 (15)
C5—C4—H4B	109.2	O2^i^—Zn1—O3^i^	88.54 (15)
H4A—C4—H4B	107.9	O3—Zn1—O3^i^	180.0
C6—C5—C4	111.7 (4)		
			
C6—C1—C2—C3	56.4 (6)	C5—C6—C7—O1	−14.3 (6)
C1—C2—C3—C4	−56.7 (7)	C1—C6—C7—O2	−69.8 (5)
C2—C3—C4—C5	57.1 (7)	C5—C6—C7—O2	167.9 (4)
C3—C4—C5—C6	−56.2 (7)	O1—C7—O2—Zn1	−13.6 (6)
C2—C1—C6—C7	−177.8 (4)	C6—C7—O2—Zn1	164.1 (3)
C2—C1—C6—C5	−53.7 (6)	C7—O2—Zn1—O2^i^	115 (24)
C4—C5—C6—C7	174.6 (5)	C7—O2—Zn1—O3	−79.1 (3)
C4—C5—C6—C1	53.1 (6)	C7—O2—Zn1—O3^i^	100.9 (3)
C1—C6—C7—O1	107.9 (5)		

Symmetry codes: (i) −*x*+1, −*y*+1, −*z*.

### Hydrogen-bond geometry (Å, °)

**Table:**

*D*—H···*A*	*D*—H	H···*A*	*D*···*A*	*D*—H···*A*
O7—H7···O1	0.83 (1)	1.99 (3)	2.699 (4)	142 (5)
O3—H3D···O1^ii^	0.85 (1)	2.53 (7)	3.132 (6)	129 (8)
O3—H3C···O7^iii^	0.85 (1)	2.17 (2)	2.997 (5)	164 (6)

Symmetry codes: (ii) *x*, −*y*+1, *z*−1/2; (iii) −*x*+1, −*y*+2, −*z*.
